# Optimal Control of Direct Contact Membrane Distillation Operated under Fluctuating Energy Source

**DOI:** 10.3390/membranes12060628

**Published:** 2022-06-16

**Authors:** Emad Ali

**Affiliations:** Chemical Engineering Department, King Saud University, Riyadh 11421, Saudi Arabia; amkamal@ksu.edu.sa

**Keywords:** water desalination, membrane distillation, fluctuating energy, automatic control, optimal

## Abstract

An optimal control strategy was tested to regulate the flow rate of the cold stream to maximize the time-averaged water production of a laboratory-scale membrane distillation (MD) process. The MD process is operated under fluctuating inlet hot temperatures at a fixed flow rate for the hot stream. The inlet hot temperature fluctuates due to fluctuation in the supplied renewable energy source, such as solar energy. The simulation revealed the possibility of enhancing the average water production by up to 4.2%, by alternating the flow rate of the cold stream relative to a fixed flow rate of the hot stream. The enhancement was limited because, when using a long membrane, the mass flux degrades when the ratio of the cold stream to the hot stream flow rates is either very high or low. By modifying the control strategy to adapt the membrane length in addition to the flow rate of the cold stream, highly improved performance could be obtained. In fact, up to 40% enhancement in the average water production was observed.

## 1. Introduction

Water is crucial for human life and nature. The demand for fresh water is soaring rapidly due to the expanding population and urbanization, while there are depleting pure water resources. It is estimated that the total demand for fresh water in the year 2000 was around 4 × 10^9^ cubic meters, and it is anticipated to have risen by at least two folds by the year 2030 [1], which will mean that humanity will face water shortage by that time [1]. Till now, desalination remains the most trusted and practical technology for supplying potable water. However, all the conventional desalination technologies, such as the multistage flash (MSF), multiple-effect distillation (MED), and reverse osmosis (RO), are energy-demanding methods, irrespective of their advantages. Therefore, the projected growth of water desalination technology must comply with reducing energy consumption and environmental footprint. It is estimated that the energy demand of the expected desalination projects will reach up to 2.4 GWh by 2030 [2]. Hence, this situation mandates the search for sustainable and energy-efficient desalination methods. Nowadays, membrane distillation (MD) technology has been trending and rapidly growing because of its appealing properties, especially the ability to be driven by low-grade energy sources [3,4,5,6,7]. Furthermore, MD can treat very highly concentrated solutions [3,4,5,6,7]. MD is a joint thermal and membrane separation process. Direct contact MD (DCMD) is the simplest and most studied variant of MD. Reports indicate versatile application of DCMD [4,8,9]. It is known for a high-gain output ratio (GOR), relative to other MD variants, if judicially tuned [10]. Nevertheless, the extensive commercialization of MD is modest, due to its known downsides. The major weaknesses of DM are fouling, membrane wetting, and low recovery ratio [3,4,5,11,12,13]. Yet, small-capacity MD plants for water production are deployed in some locations [4,14]. Despite the recent commercialization of low-capacity MD systems, further investigations are necessary to promote its widespread and large-scale application [6,14]. Another issue related to MD is its high specific energy demand, which is around 39–60 kWh/m^3^ [15].

To tackle these aforementioned shortcomings of MD, numerous and diverse studies that cover versatile aspects of MD technology were reported in literature. The goal is to introduce MD as a cost-effective and trustworthy desalination technology to compete with existing methods. For instance, the implementation of the MD process in the treatment of brackish water and seawater has been widely investigated, both experimentally and theoretically [4,5,11,12,16,17]. Other studies cover integrating the MD modules with heat recovery devices [10,18,19], recycling brine to amplify the recovery ratio [18,19], and employing the multistage concept [19,20]. Other researchers have studied the integration of MD with low-grade energy resources [21,22]. In fact, the latter studies consider powering the DM with renewable energy sources to overcome the high specific energy demand. However, renewable energy is known for its fluctuation and intermittency. For example, solar energy is limited during the night time and/or cloudy weather.

Some researchers started focusing on the transient modeling and temporal analysis of MD processes [23,24,25,26]. Ali et al. [27] and Ali et al. [28] studied different complexities of the unsteady-state models. Nevertheless, most of the reported works in this regard dealt only with developing, validating, and analyzing the transient behavior. To the author’s knowledge, no study has reported dealing with utilizing the MD dynamic model to test an automatic control system, and/or analyzing the effect of fluctuating disturbances on the temporal behavior of the MD process. It is of interest to utilize the dynamic model to enhance MD operation and temporal performance. For example, one can design optimal feed trajectories to counter the impact of fluctuating and/or intermittent energy sources. For instance, operating a reverse osmosis desalination unit periodically resulted in enhanced mass flux [29]. This was attributed to the fact that a fluctuating flow rate induces turbulence that reduced the concentration polarization and fouling [30,31,32]. Gustafson et al. [33] investigated the impact of an intermittent energy source on MD performance. However, their investigation focused on the process structure using a static model. Generally, the steady-state analysis overlooks the dynamic variation of the performance due to the inherent internal transient lag of the process. Ignoring such an effect can cause the inadequate design of power systems, such as those based on solar and wind energies, which are characterized by temporal behavior. 

Operating the MD process using renewable energy is challenging because renewable energy, such as solar or wind energy, is fluctuating and intermittent. Solar energy, in particular, is preferable for powering MD because it can be directly provided as thermal energy. Any disturbance in the solar energy source deteriorates the MD performance as thermal energy is the main driver for the separation process. Researchers have dealt with the intermittency and fluctuation of renewable energy through three approaches [31]. One approach is to integrate solar and wind energies such that a continuous supply of energy can be maintained [34,35,36,37]. However, this will make the system complicated and requires sophisticated power management systems to optimally alternate between the two sources. Another approach dealt with aiding the renewable energy source with an energy storage system, i.e., battery banks [32,38]. This approach also requires a power management system and advanced instrumentation. Furthermore, proper sizing and maintenance of the battery banks are necessary. The third approach deals with manipulating the process operation by utilizing advanced control systems and/or artificial intelligence systems. The proposed strategy in this work falls within the third approach. It is not meant to overrule or replace the other strategies. It can be applied with and without the other strategies to provide possible enhancement in the process operation during disturbances in the energy source. Hence, the objective here is to design and test an optimal control strategy to maintain the maximum production rate during thermal energy fluctuation. This can be achieved by dynamically manipulating the flow rate of the cold stream in the MD unit. It is believed that a high ratio of the flow rate of the cold stream relative to the hot stream can improve the mass flux in the MD unit [39]. Most reported studies have dealt with fixed and equal flow rates for the hot and cold streams. In this work, the cold stream flow rate will be time-varying as governed by the optimal control strategy.

## 2. Dynamic Model of the MD Process

**Figure 1:**
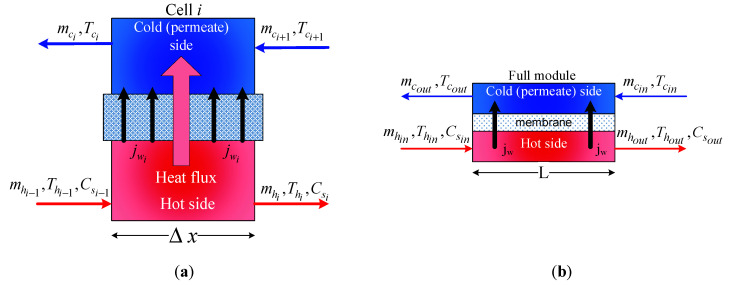
Schematic of the MD process: (**a**) control volume and (**b**) whole module.

To study the effect of alternating energy sources and to investigate the effectiveness of the proposed control system, a dynamic model for the MD process was required. For this purpose, a previously developed and validated time-evolution model of the DCMD unit [27,28,40] was used here. Hence, the scope here is not to develop a new model or modify an existing one, but rather to present the dynamic model needed for optimal control. Note that the optimal control is a model-based method that uses the model to predict the future behavior of the process. To avoid repetition, a brief description of the unsteady-state model is explained. Essentially, the transient model was generated by expressing the unsteady-state heat balance around a control volume (Figure 1a) of the MD module. Assuming the full MD module (Figure 1b) consisted of *n* homogeneous and equally sized control volumes, the obtained mathematical equations for the entire cells (control volumes) could be as follows [27,28,40]:

(1) $$\left(\frac{v}{n}\right)\rho Cp\frac{dT_{h_{i}}}{dt}=m_{i}Cp\left({T_{h}}_{i-1}-T_{h_{i}}\right)-\left(j_{w_{i}}h_{v_{i}}+h_{m_{i}}\left(T_{h_{m,i}}-T_{c_{m,i}}\right)\right)\Delta xl$$

(2) $$\left(\frac{v}{n}\right)\rho Cp\frac{dT_{c_{i}}}{dt}=m_{i}Cp\left({T_{c}}_{i+1}-T_{c_{i}}\right)+\left(j_{w_{i}}h_{v_{i}}+h_{m_{i}}\left(T_{h_{m,i}}-T_{c_{m,i}}\right)\right)\Delta xl$$

Assuming a pseudo-steady state (PSS), the water and salt balance could be written as follows:

(3) $$m_{h_{i}}={m_{h}}_{i-1}-m_{w_{i}}$$

(4) $${m_{c}}_{i+1}=m_{c_{i}}+m_{w_{i}}$$

(5) $$C_{s_{i}}={C_{s}}_{i-1}{m_{h}}_{i-1}/m_{h_{i}}$$

$$\begin{array}{l} \;(i=1,\ldots ,n)\\ for\;i=n\to T_{h_{i}}\equiv {T_{h}}_{out}\;;{T_{c}}_{i+1}\equiv {T_{c}}_{in} \end{array}$$

$$for\;i=1\;\to {T_{h}}_{i-1}\equiv {T_{h}}_{in}\;;T_{c_{i}}\equiv {T_{c}}_{out}\;;{m_{h}}_{i-1}={m_{h}}_{in}\;;\;m_{c_{i}}={m_{c}}_{in};{C_{s}}_{i-1}=C_{s_{f}}$$

Note that $m_{h_{in}},\;m_{c_{in}},\;T_{h_{in}},\;T_{c_{in}},\;and\;C_{s_{in}}$ were the process inputs while $T_{h_{out}},T_{c_{out}},\;and\;m_{w}$ were the process outputs. The feed salinity ($C_{s_{in}}$) was taken as 3000 ppm to simulate typical brackish water. The inlet cold feed temperature ($T_{c_{in}}$) was fixed at 20 °C. Thereby, the process has three independent inputs, namely $m_{h_{in}},\;m_{c_{in}},\;and\;T_{h_{in}}$, that can manipulate the process behavior and performance. In this study, $m_{h_{in}}$ was fixed, while $and\;T_{h_{in}}$ were able to vary due to the fluctuating nature of the energy source. This made $m_{c_{in}}$ the available manipulated variable. The given transient model was solved numerically by the Euler method with the aid of MATLAB software. For all numerical simulations, the number of cells, n, was set to 10 and the integration step size was set to 10 s, which was good enough to sustain a stable numerical solution. The proposed transient model was previously validated against experimental data. Indeed, the outlet temperatures were verified by Ali et al. [28,40], while the mass flux was validated by Ali et al. [27]. The point here was to utilize the authenticated models in investigating the effectiveness of the control system to improve the process performance during disturbances in the energy sources. 

The numerical solution of the time-evolution model involved several intermediate parameters, such as $j_{w},\;h_{v},\;h_{m},\;T_{h_{m}},\;and\;T_{c_{m}}$, at each time step. These local variables were obtained by iteratively solving the joint mass and heat-transfer equations, as described in Appendix A and in [27,28,40]. The underlying mass and heat-transfer equations, presented in Appendix A, implicitly relied on the membrane sheet characteristics. The membrane characteristics in this study were as follows: the effective area was 10 m^2^, the thickness was 230 μm, the channel length was 14.3 m, the channel height was 0.7 m, the pore diameter was 0.2 μm, and the porosity was 0.8. The membrane characteristics were based on the experimental module used to validate the model [28,40]. 

## 3. Control Strategy

**Figure 2:**
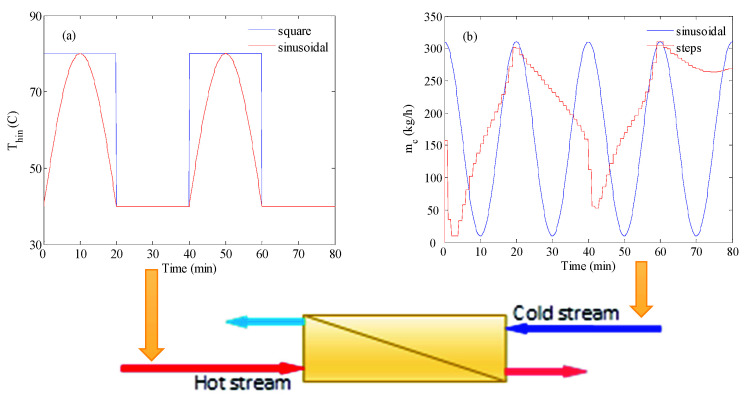
Input signals applied to MD; (**a**) hot feed temperature variation with solar energy; (**b**) inlet cold flow rate regulated via control system.

The typical MD unit is driven by the thermal energy associated with the feed stream. The feed thermal energy can be natural, i.e., geothermal, or a utility stream in a typical industrial plant. Alternatively, it can be obtained from an external energy source. To reduce the cost of energy consumption, thermal energy can be obtained from low-grade energy or solar energy. The latter is known for its fluctuation from daylight to night and from sunny day to cloudy day. Gustafson et al. [33] represented this variation by a square wave. Najib et al. [41], studied the use of solar energy to power the VMD process. Their measurement of the solar-heated water showed a semi sinusoidal variation of the water temperature during the day. The solar intensity and, consequently, the water temperature started rising slowly from the early morning till it reached a maximum around noon. Afterwards, they gradually declined till sunset, where the water temperature became asymptotic. A typical representation of the inlet hot temperature could be used, as shown in Figure 2a. The hot temperature variation, due to fluctuation in the solar energy source, could be represented in the study either as a square wave or pseudo-sinusoidal function. Both functions had a period of 40 min. This means that during half of the time interval, the solar energy was active and during the other half it was inactive. This would represent the natural behavior during a typical day. However, a shorter time scale was used here to match the dynamic characteristics of the MD model, which represented a laboratory-scale unit. Of course, for an industrial scale, different time scales could be used. Different representations of the energy fluctuation could be considered. However, this was enough to test the proposed control strategy.

Manipulation of the inlet cold stream in real-time required a typical feedback control system. In this study, we studied the use of an optimal control strategy, which determines the optimal input trajectory that maximizes a cost index. In this study, the cost index was taken as the average production rate. The optimal control strategy could be formulated as follows:

(6) $$\underset{m_{c}\left(t_{k}=0\right),\ldots m_{c}\left(t_{k}=t_{f}\right)}{\max}\emptyset =\frac{\int_{0}^{t_{f}}m_{w}\left(t_{k}\right)}{\int_{0}^{t_{f}}t}$$

Subject to:

(7) $$80\leq m_{c}\left(t_{k}\right)\leq 1000;\;\;\;\;\;\;\;\;t_{k}=t_{0},\;t_{1},\ldots t_{f}$$

According to Equation (6), the optimal strategy was set to maximize the average production rate over a simulation time interval $t_{f}$. The latter was set to 80 min in this study. The maximization was achieved by determining the optimal values of $m_{c}\left(t_{k}\right)$ over the simulation interval. The trajectory of *m_c_* was set as a series of steps. Each step was bounded between 80 and 1000 kg/h. The number of these steps depended on the sampling time. Different values for the sampling time were tested. A typical representation of the series of steps is shown in Figure 2b, by the red color. In this case, the controller had a large degree of freedom, i.e., a large number of steps over the simulation time. However, the numerical simulation of the optimization problems became computationally intensive. Another way to design the input trajectories of *m_c_* was to formulate them as sinusoidal (series of parabolic pulses), as shown in Figure 2b, by the blue color. To ensure positive values for the input trajectory the parabolic pulses could be expressed mathematically as follows:

(8) $$m_{c}\left(t\right)=a\times \left[\sin\left(\frac{2\pi t}{T}\right)+1\right]+10$$

Which would be denoted as a sinusoidal function. Another representation of the pulses was as follows:

(9) $$m_{c}\left(t\right)=a\times {\left(\sin\left(\frac{2\pi t}{T}\right)\right)}^{2}+10$$

The second formulation made the pulses sharper and would be denoted as squared sinusoidal. Hence the optimal control strategy using the pulses trajectories became:

(10) $$\underset{a,T}{\max}\emptyset =\frac{\int_{0}^{t_{f}}m_{w}\left(t_{k}\right)}{\int_{0}^{t_{f}}t}$$

Subject to:

(11) 10≤a≤350

(12) 2≤T≤60

In Equation (10), the objective function was defined as the average production rate over a specific time interval of *t_f_*. In this case, the number of design parameters was reduced to two, i.e., the pulse amplitude (*a*) and the period (*T*). Note that the amplitude and the period were bounded to fit the operational limits. It should be noted that it was not the purpose here to develop a new control strategy or test the effectiveness of a certain control algorithm. Instead, it was desired to determine input time-trajectories for the permeate flow rate to improve the mass production during fluctuation in the inlet feed temperature due to fluctuating energy sources. One way to deal with this issue was to cast the problem as an optimal control problem. Classical control systems, such as proportional-integral-derivative, determine the current-time control action (input) based on the current process measurement. An optimal control strategy, however, could predict the future input trajectories (control actions) using the dynamic model. Moreover, controllability and practical implementation issues were not considered here. The objective here was to assess the concept of alternating the flow rate ratio to create disturbance inside the membrane sheet and, subsequently, to produce more pure water.

In general, unsteady change in the cold stream flow could cause profound impact on the mass flux, as discussed in previous work [42]. In fact, step changes in *m_c_* relative to *m_h_* resulted in a nonlinear effect on the static gain and time constant of the process. Thereby, frequent and rapid changes in *m_c_* could cause non-homogeneous distribution of the bulk temperature, as well as temperature polarization along the module length. This, in turn, would improve the performance in terms of the mass flux.

## 4. Results and Discussion

### 4.1. Process Behavior

**Figure 3:**
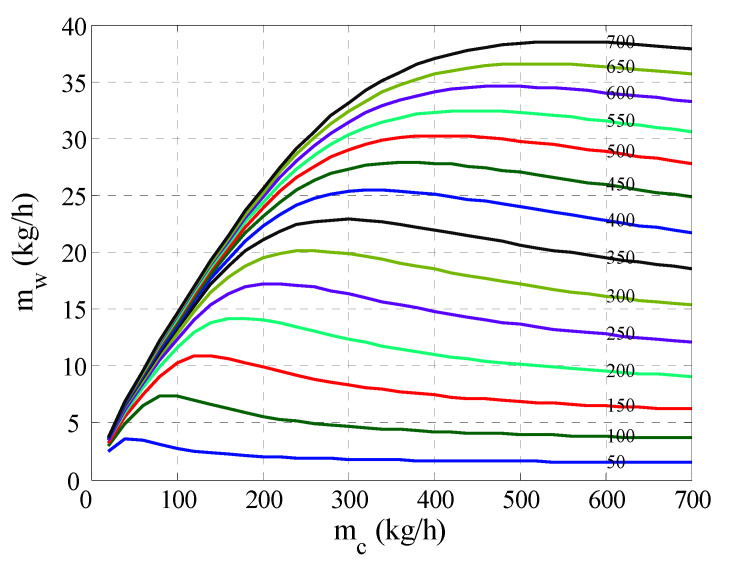
Variation of production rate with feed and permeate flow rates at $T_{h_{in}}=80\;{}^{\circ}C$ and $T_{c_{in}}=20\;{}^{\circ}C$. Different colored lines corresponds to different $m_{h}$ values as indicated on the curve itself.

Simulation of the existing model for various values for the feed and permeate flow rates at a fixed inlet hot temperature of 80 °C, a fixed inlet cold temperature of 20 °C, and a fixed length of 14.3 m was conducted. The result is shown in Figure 3. Evidently, the water mass production (*m_w_*) increased with flow rates which is well-known behavior for MD. In almost all cases, the water mass flux reached its maximum value when both flow rates were almost equal. In fact, the maximum flux occurred roughly when the ratio $m_{c}/m_{h}$ was 80%. For all values of feed flow rate, the mass flux trend exhibited a steep increase at the lowest values for the permeate flow rate. After passing the maximum value, the mass flux decreased slowly with permeate flow rate. At a high flow rate ratio, i.e., $\frac{m_{c}}{m_{h}}\gg 1$, the abundance of cold stream quenched the hot stream readily and considerably at the first half of the module length, i.e., toward the entrance of the cold stream. This created a very narrow bulk temperature difference at the first half of the module length. On the other half of the module, towards the hot stream inlet, the bulk temperature difference became much wider, because the hot stream’s temperature at the entrance was highest, while the temperature of the cold stream was not warm enough. Note that the cold stream did not get much warmth because of its high capacitance induced by its high mass rate. As a result, the average bulk temperature difference became smaller than that at $\frac{m_{c}}{m_{h}}\approx 1$. Subsequently, the water mass flux would become proportionally smaller. On the other hand, at a very low flow rate ratio, i.e., $\frac{m_{c}}{m_{h}}\ll 1$, a similar, but more severe, phenomenon occurred. When the cold stream had a minute flow rate relative to the hot stream, it became hot till it approached the hot stream temperature at the location where the hot stream was fed. At the other end, where the cold stream was fed, the cold stream became warm, because the hot stream still possessed high thermal capacitance. This situation created a very narrow bulk temperature difference along the entire MD module and, consequently, negligible mass flux. Ali et al. [14] have also reported that positive bulk temperature difference cannot be maintained for a long MD module. Note that the internal effect of $R_{m}=\frac{m_{c}}{m_{h}}$ on the temperature distribution along the membrane length, and its impact on the hydrodynamic and temperature polarization, is discussed in earlier work [42]. In conclusion, alteration of the flow rate relative to each other caused a remarkable effect on the water mass flux and, hence, on the production rate.

**Figure 4:**
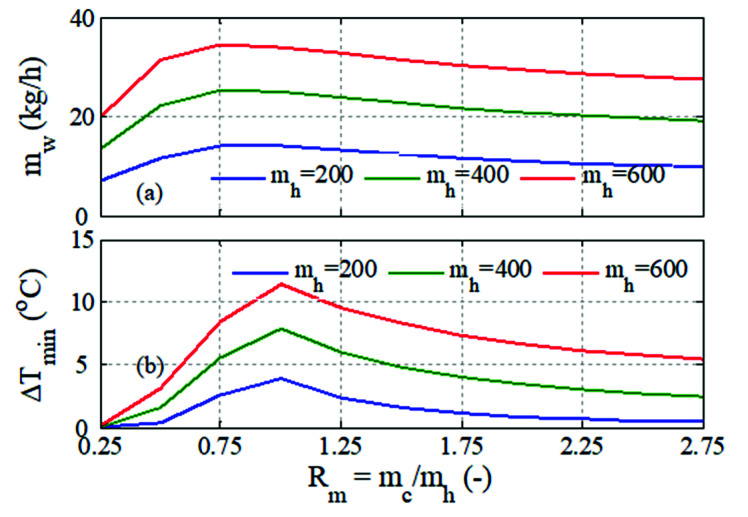
Effect of flow rate ratio on the steady state water production and minimum temperature difference at $T_{h_{in}}=80\;{}^{\circ}C$; (**a**) water production, (**b**) minimum temperature difference.

Figure 4 further explains the effect of varying the cold stream flow rate with respect to the hot stream flow rate, i.e., the ratio of $m_{c}/m_{h}$. The result showed the performance at three fixed values for *m_h_*. Here we focused on the overall influence of the variant flow rate on the MD output, which was more relevant to the analysis in this study. Figure 4a depicts the variation of mass production at a steady state with an increasing flow rate ratio. The water production increased with R  till it reached asymptotic behavior, which is the same as that shown in Figure 3. However, the range for $m_{c}$ was wider and varied with the value of $m_{h}$. For example, for $m_{h}=600\;\mathrm{kg}/h$, the range of $m_{c}$ fell between 150 and 1650 kg/h. The behavior of *m_w_* with *R_m_* could be explained by the result shown in Figure 4b, which displays the variation of T_min_ with *R_m_*. T_min_ was the minimum difference between the hot and cold bulk temperatures along the membrane length. In the standard case, i.e., *R_m_* = 1, the difference between the hot and cold temperatures was almost constant over the length of the membrane. In fact, it exhibited the maximum value, as shown in Figure 4b. For low *R_m_*, less cold permeate was circulated inside the membrane, hence the cold stream became overheated till its outlet temperature approached the hot temperature, causing T_min_ to approach zero. In this case, the temperature difference became very narrow on the membrane side where the hot stream was fed and wider on the other side. As a result, *m_w_* became very small, as shown in Figure 4a. For the given membrane length of 14.3 m in this study, no negative mass flux was detected. At high *R_m_*, excess cold permeate was circulated, which absorbed most of the hot stream thermal energy. As a result, the outlet brine temperature approached the inlet permeate temperature (${T_{c}}_{in}$), and, hence, T_min_ gradually declined_._ In this case, the temperature difference became narrower on the membrane side where the brine left the unit, but much wider on the other side. Although T_min_ decreased at high *R_m_*, *m_w_* remained high, because the average bulk temperature difference along the membrane length remained high. Interestingly, the maximum water production did not occur at *R_m_* = 1 where T_min_ was at a maximum. Although the bulk temperature difference was an indicator of the mass flux capacity, the latter actually depended on the temperature difference at the membrane interface ($T_{h_{m}}-T_{c_{m}}$), which was strongly dependent on the film heat transfer of each channel. The latter was influenced by the corresponding flow rate in each channel. Moreover, the permeability coefficient, $C_{m}$ which was also a function of the average of the membrane-surface temperatures, affected the mass flux across the membrane. The combinatorial effect of these parameters made the maximum production occur at *R_m_* ≈ 0.8.

**Figure 5:**
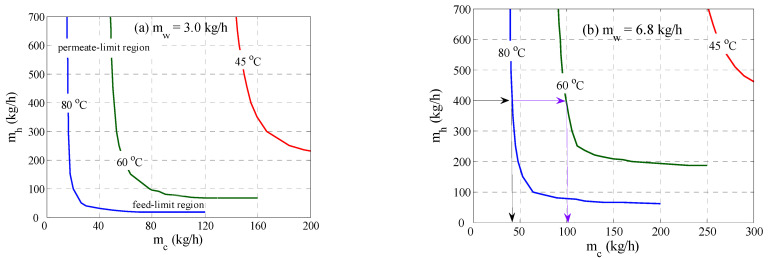
Operation range for different values for the inlet feed temperature.

To further assess this behavior, the operation limit was generated as shown in Figure 5. The operability curves were obtained by fixing the mass flux to a target value and determining the combination of feed and permeate flow rate that led to the same target mass flux. The procedure was repeated for selected values for the inlet feed temperature namely; 80, 60, and 45 °C. The operation lines displayed a convex behavior that became less severe at lower hot feed temperatures. Clearly, the operating range for the permeate flow rate increased as the required production rate grew, as can be seen by comparing Figure 5a with Figure 5b. Moreover, at a low permeate flow rate, a very large feed flow rate was needed to achieve the same mass flux. In this case, the operation became permeate-limited. In this region, adjustment of the heat and mass transfer in the hot channel, by altering its flow rate, would cause a negligible impact on the process performance. Similar observation could be analyzed at a low hot stream flow rate, where the process became feed-limited. Nevertheless, if we considered operating at a feed flow rate of 400 kg/h and inlet temperature of 80 °C, then, as shown in Figure 5b, the permeate flow rate had to be approximately 40 kg/h to maintain the production rate of 6.8 kg/h. If the inlet feed temperature suddenly dropped to 60 °C, due to disturbances in the energy source, then the cold stream flow rate had to be increased to approximately 100 kg/h to maintain the same production rate. This meant a proper automatic control system was needed to online regulate the permeate flow rate to achieve the desired operation during disturbances and/or alternating energy sources.

### 4.2. Optimal Control Analysis

**Figure 6:**
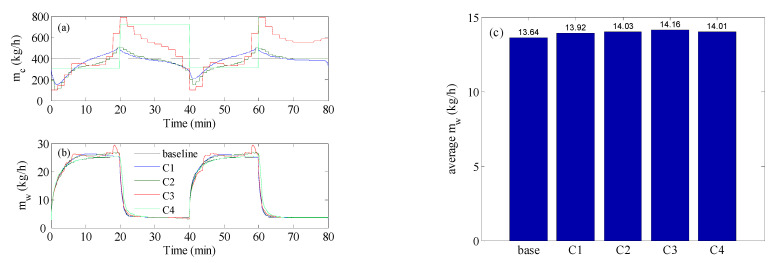
Optimal control result when the inlet temperature varies as a square wave; (**a**) cold stream flow rate, (**b**) mass production rate, (**c**) Time-averaged mass production; C1: T_s_ = 20 s, C2: T_s_ = 1 min, C3: T_s_ = 2 min, C4: T_s_ = 20 min. $m_{h}$ is fixed at 400 kg/h.

As shown in Figure 5, when the feed temperature changed due to fluctuations in the energy source, the production rate would deplete. For this purpose, the proposed control strategy was tested to produce enhancive control actions. The test of the control strategy to regulate the process during inlet temperature fluctuation is shown in Figure 6. In this case, the inlet temperature of the hot feed was alternating in a square waveform, as is shown in Figure 2a. The optimal control would adjust the cold stream as a series of steps like that shown in Figure 2b. For the base case, the hot and cold flow rates were fixed at 400 kg/h, as depicted by the dashed line in Figure 6a. The corresponding response of the mass production for the baseline case alternated as shown in Figure 6b, because the inlet temperature was fluctuating. In fact, it followed the same trend as the inlet temperature. As expected, *m_w_* reached a high value when the inlet temperature was high, i.e., 80 °C, and vice versa. Obviously, a loss of production took place when the inlet temperature was low because of the lack of solar irradiation. Hence, the average value of *m_w_* over the operation period would be lower than that at 80 °C. Therefore, the objective of the optimal control was to enhance the overall performance, in the sense of maximizing the average value of *m_w_* over the operating period, by manipulating the cold stream flow rate, *m_c_*. The optimal control was implemented at different sampling times of 20 s, 1 min, 2 min, and 20 min, and the results are displayed in Figure 6. Using a small sampling time would generate a larger number of control actions (cold stream flow trajectories). Hence, the control system would have a larger degree of freedom and, subsequently, a better opportunity to enhance the performance. As demonstrated in Figure 6a, the control system managed to generate alternating *m_c_* for all cases of sampling times. For all tested sampling times, since the control objective was to maximize the average value of $m_{w}$, the controller forced *m_c_* to grow when the inlet temperature was low. This is a rational result that coincides with the finding of Figure 4a, which mandated increasing the flow rate ratio to increase the mass production. Interestingly at T_s_ of 20 min, which was equivalent to the half of the period of the square wave of the inlet temperature, the generated profile of *m_c_* resembled a full square wave that counteracted that of the inlet temperature, i.e., *m_c_* was low when $T_{h_{in}}$ was high, and vice versa. The time response of the water production rate that corresponded to *m_c_* trajectories is depicted in Figure 6b. Although the *m_w_* responses were overlapping, apparent changes could be detected during the period when $T_{h_{in}}\;$ was high (80 °C). However, minor changes occurred when the inlet temperature was low (40 °C). The minor changes were obscured by the plotting scale. The minimal changes could be ascribed to the fact that improving the heat and mass transfer, via flow alteration, had a minor effect at a low feed temperature of 40 °C. At this low value, the temperature drop along the MD length was minor, which made the bulk temperature very narrow for a long module. Thereby, less effective temperature polarization distribution could be generated by flow alteration. To assess the effect of the optimal control, the time-averaged value of *m_w_* was computed and compared in Figure 6c. For all tested values for T_s_, minor enhancement in the average *m_w_* was observed over the baseline case. The maximum improvement occurred at T_s_ = 2 min with an average *m_w_* of 14.16 kg/h, which corresponded to 3.7% enhancement. Besides achieving slight improvement, most of the enhancement occurred during the high inlet hot temperature. This meant the controller failed to improve the performance during the absence of solar irradiation.

**Figure 7:**
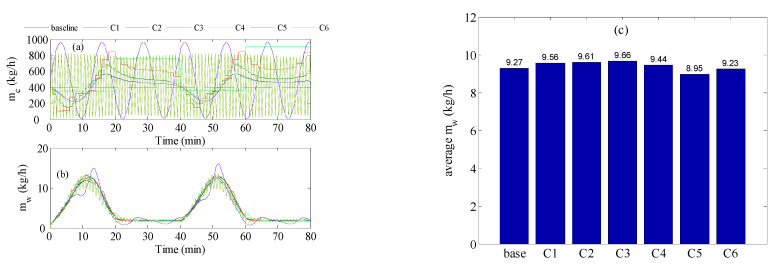
Optimal control result for inlet temperature varies as a sine-like wave; (**a**) cold stream flow rate, (**b**) mass production rate, (**c**) Time-averaged mass production; C1: T_s_ = 20 s, C2: T_s_ = 1 min, C3: T_s_ = 2 min, C4: T_s_ = 20 min, C5: sinusoidal. C6: squared sinusoidal.

We reexamined the control strategy, but when the inlet temperature varied as a sine-like wave, as shown in Figure 2a by the red curve. This resembled the natural behavior of solar irradiation during the day. The optimal controller could alternate the cold stream as a series of steps or as a sinusoidal function, as depicted in Figure 2b. The result of the simulation is illustrated in Figure 7. The figure shows the results using repeated steps and sinusoidal functions. This simulation was very similar to that conducted in Figure 6 except that a more realistic profile for the inlet hot temperature was utilized. Figure 7a shows the control actions (cold stream flow trajectories) generated by the optimal control. When the control action was cast as repeated steps, cold stream flow trajectories similar to that obtained in Figure 6a were generated. In the other case, the optimal control strategy generated sinusoidal control actions. Figure 7b demonstrates how the resulted *m_w_* response for all cases behaved as a sine-like function, because the inlet hot temperature varied in the form of a sine-like function. Assorted variations in the *m_w_* response were observed, due to the different trajectories of *m_c_* generated by the optimal controller. When *m_c_* was allowed to change as a sinusoidal function it delivered the worst performance, especially for the squared sinusoidal function. The latter exhibited very oscillatory dynamic behavior. This could be further assessed by examining Figure 7c which compares the average *m_w_* over the period for all cases. There is no doubt that the sinusoidal case (C5 & C6) had the worst performance, manifested by *m_w_* value lower than that of the baseline. Once again, the steps-like function, with a sampling time of 2 min, delivered the best performance with an average *m_w_* equal to 9.66 kg/h which was equivalent to 4.2%. Nevertheless, the enhancement gained by using the optimal controller was still marginal. The inferiority of the sinusoidal representation of *m_c_* was ascribed to its low degrees of freedom. The sinusoidal function had only 2 parameters to tune, which were the amplitude and the period of the periodic function. On the other hand, the steps-like representation had a higher number of degrees of freedom, which varied from 240 for T_s_ = 2 s to 4 for T_s_ = 20 min. Nevertheless, the improvement made by the proposed control strategy was still limited and its contribution during low solar irradiation was still minimal. Another reason for the limited contribution of the control strategy during low solar irradiation/temperature was the formulation of the control objective in Equations (6) and (10). The objective function was a lumped average value of the water produced during the high and low intervals of solar irradiation. In this case, the ample value of $m_{w}$ during high-temperature intervals overweighed that at low-temperature intervals which prevented the control strategy making the expected corrective actions.

### 4.3. MD Length Retrofitting

**Figure 8:**
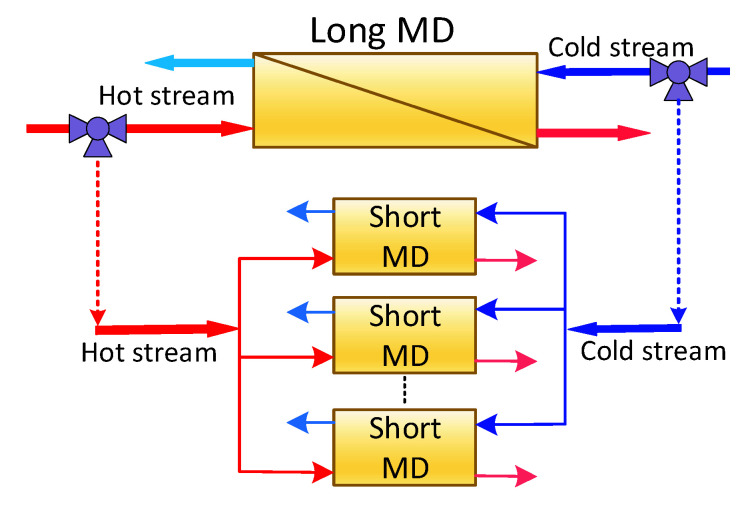
Short length MD configuration.

As mentioned earlier, the control strategy failed to improve the MD performance substantially, especially during the period when the inlet temperature was low. This could be attributed to the membrane length. As Figure 4 indicates, for a fixed MD length at a nominal value of 14.3, high, and, more particularly, low flow rate ratio, could generate little mass flux. In this case, more than half of the membrane length was not leveraged to separate more pure water. In other words, adjustment of the heat and mass transfer was ineffective for a long membrane with a low flow rate ratio. Therefore, it was of interest to adapt the membrane length at low/high flow rate ratios to maintain reasonable temperature differences along the MD module. Maintaining reasonable temperature difference resulted in a higher mass flux. For this purpose, we proposed the structure shown in Figure 8.

In due course, instead of using a long MD module, multiple short modules arranged in parallel could be implemented. For integrity, the sum of the length of all short modules should be equal to the nominal long module. Note that the corss sectional area remained the same, such that the total surface area of the short modules and the long m odule remained unchanged. Since the cost of the MD module was based on its surface area (length), the two configurations would have almost the same cost. Moreover, for a fair comparison with the long module, the same total flow rate should be used for the parallel structure. In this case, the original feed flow rates should be split into equal streams to be fed to the individual parallel MD units. Note that, since the cross sectional area was fixed, the haydraulic would be affected and so would the heat transfer coefficient. This structure should be active when the flow rate ratio was low and/or the minimum temperature difference was below a certain threshold. A three-way valve could be used to switch between the two configurations. Switching between long and short module configurations was not easy in practice. However, the opinion here was to assess the feasibility of this concept. To adapt the MD length online, the optimal control law was modified as follows:

(13) $$\underset{m_{c}\left(t_{k}=0\right),\ldots m_{c}\left(t_{k}=t_{f}\right),\;ln}{\max}\emptyset =\frac{\int_{0}^{t_{f}}m_{w}\left(t_{k}\right)}{\int_{0}^{t_{f}}t}$$

Subject to:

(14) $$80\leq m_{c}\left(t_{k}\right)\leq 1000;\;\;\;\;\;\;\;\;t_{k}=t_{0},\;t_{1},\ldots t_{f}$$

(15) 1≤ln≤14.3

(16) $$\Delta T_{min}\geq 0.5$$

**Figure 9:**
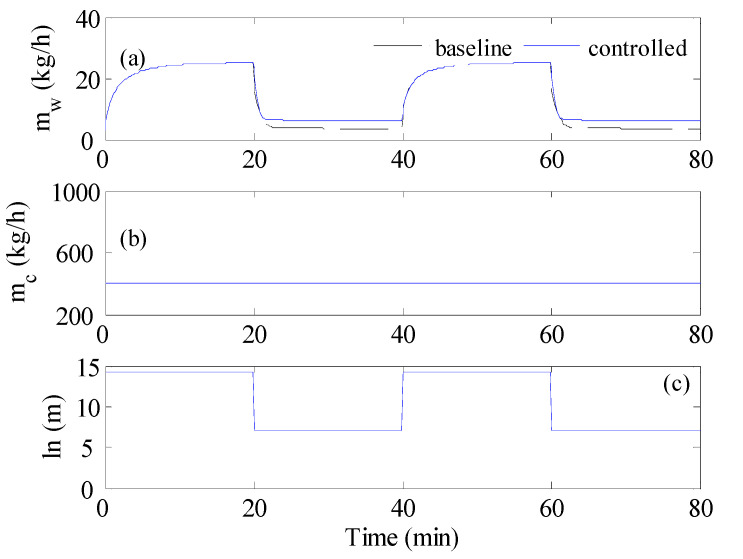
Optimal control results with active constraints for square wave inlet hot temperature, $m_{h_{in}}=400\;\frac{\mathrm{kg}}{h};T_{h_{in}}=80\;{}^{\circ}C$, (**a**) Productivity, (**b**) cold stream flow rate, (**c**) module length.

**Figure 10:**
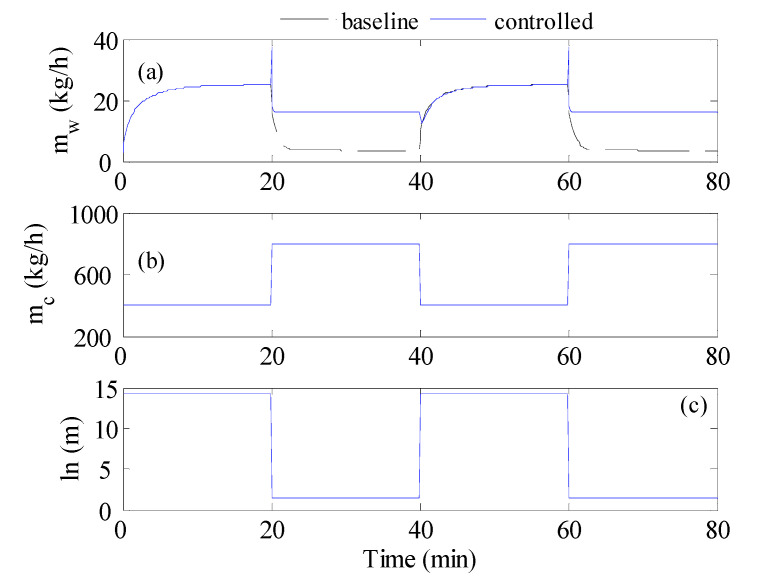
Optimal control results without constraints for square wave inlet hot temperature, $m_{h_{in}}=400\;\frac{\mathrm{kg}}{h};T_{h_{in}}=80\;{}^{\circ}C$, (**a**) Productivity, (**b**) cold stream flow rate, (**c**) module length.

**Figure 11:**
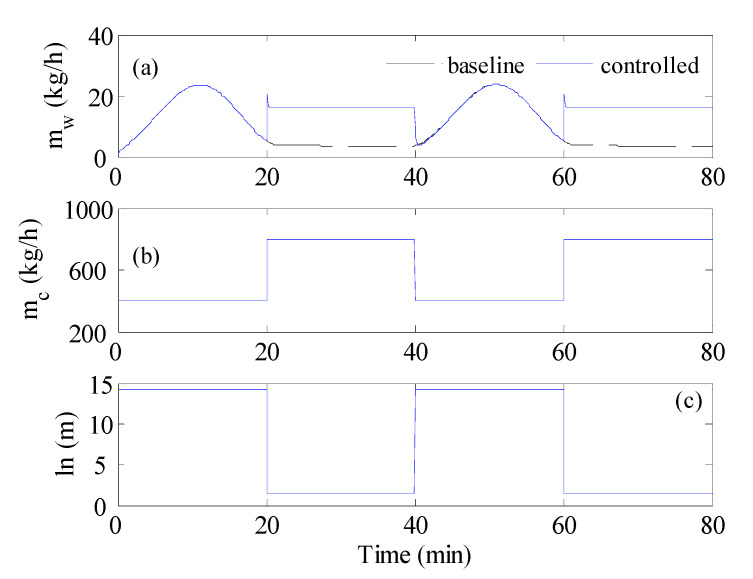
Optimal control results without constraints for sine-like wave inlet hot temperature, $m_{h_{in}}=400\;\frac{\mathrm{kg}}{h};T_{h_{in}}=80\;{}^{\circ}C$, (**a**) Productivity, (**b**) cold stream flow rate, (**c**) module length.

In this case, the control strategy involved the length of the membrane (*ln*) as a design parameter. To preserve physical limits, the MD length was bounded between 1 and 14.3 m. In addition, the optimal control law involved additional nonlinear constraints on the minimum temperature difference. A value of 0.5 °C was set as the acceptable minimum temperature difference. The purpose of the nonlinear constraint was not only to ensure reasonable temperature difference but also to force the control law to reduce the membrane length, searching for conditions that might enhance the mass flux. The optimal control would be activated only when the inlet temperature was low, i.e., 40 °C. The modified control strategy was applied to the system when the inlet hot temperature varied as a square wave. The result is depicted in Figure 9. The response of $m_{w}$ shown in Figure 9a displayed some improvement during the low $T_{h_{in}}$ period. This improvement was equivalent to 8% and was obtained by keeping the flow rate of the cold stream equal to the nominal case of 400 kg/h, i.e., a flow rate ratio of 1 (Figure 9b). Hence, the enhancement came from reducing the membrane length to half, as shown in Figure 9c. In this case, 2 short MD modules in parallel were used instead of a long one. This situation led to slight growth in $m_{w}$. The optimal control avoided increasing or decreasing the flow rate ratio because that would result in the temperature profile inside the membrane to violate the constraints set by Equation (16). Thereby, the simulation was repeated with the nonlinear constraint being disabled. The corresponding result is illustrated in Figure 10. In due course, substantial enhancement in $m_{w}$ that reached 30% over the baseline was obtained, as shown in Figure 10a. This was achieved by reducing the membrane length to 1 m, as demonstrated in Figure 10c. This was also associated with an increase in the flow rate ratio manifested by a higher $m_{c}$ flow rate, as shown in Figure 10b. Note that the flow rate of the cold stream doubled in the 20–40 min interval and at the 60–80 min interval. The optimal controller was able to increase $m_{c}$ freely because there was no restriction imposed on the minimum temperature difference. This situation helped considerably in improving the performance. The time response of $m_{w}$ in Figure 10a shows spikes at the transition points when the inlet temperature and flow rate suddenly changed. When both the MD length and flow rate suddenly changed, they caused disturbances that created momentarily non-homogenous temperature distribution along the membrane sheet. This situation led to a sudden and temporal increase in the average value of the mass flux. A similar successful result could be obtained when implementing the modified optimal control to the MD when the inlet hot temperature alternated in a sine-like profile. The result is depicted in Figure 11. Apparently substantial improvement in $m_{w}$ was obtained which amounted to 40% over the baseline. This achievement was also obtained by alternating the MD length between 1 and 15 m and the flow rate of the cold stream between 400 and 800 kg/h. In fact, the optimal controller managed, as shown in Figure 10 and, to move the design parameters, i.e., $m_{c}$ and ln in the right direction. For example, $m_{c}$ increased which would favor the growth of $m_{w}$ as indicated by Figure 4a and Figure 5. Furthermore, *ln* decreased which would restore reasonable distribution of the temperature difference at high flow rates. This would result in a reasonable average mass flux. Bearing in mind that multiple short MD modules would be utilized to resemble the long module, the resulted total mass production would be greater. In other words, the overall enhancement resulted marginally from enhancing the mass flux of the short unit by avoiding zero bulk temperature difference and largely from using multiple units. The latter was possible due to the use of shorter modules.

## 5. Conclusions

An optimal control strategy was proposed to operate the MD unit under the influence of varied inlet hot temperatures. The inlet hot temperature varied according to fluctuating energy sources, such as solar irradiation. The fluctuating solar energy was simulated in two forms; a square wave and a sine-like wave. The optimal control was formulated to maximize the time-averaged water mass production. The design parameter was taken as the input trajectories of the flow rate of the cold stream over the entire period of the simulation. Two forms of the input trajectories were simulated, namely a series of steps and a sinusoidal function. The simulation revealed the ability of the optimal control to adapt the flow rate of the cold stream to achieve minor improvement in water production. The maximum enhancement could reach 4.2% when the input trajectories were represented in the form of a series of steps using a sampling time of 2 min. Further performance improvement could be attained by modifying the control strategy to adapt the MD membrane length in addition to the flow rate. In this case, multiple short MD modules in parallel were used to restore the original MD membrane length. Implementing the modified strategy, a maximum of 40% enhancement in mass production could be obtained when the input trajectories were represented as a series of steps and the inlet hot temperature as a sine-like function.

## Data Availability

Data is contained within the article.

## Nomenclature

A	Cross-sectional area, m^2^
*C_m_*	Permeability coefficient, kg/m^2^sPa
$C_{m}^{k}$	Knudsen mass flux coefficient, kg/m^2^sPa
$C_{m}^{d}$	Molecular diffusion mass flux coefficient, kg/m^2^sPa
$C_{m}^{C}$	Transition mass flux coefficient, kg/m^2^sPa
*C*_p_	Heat capacity, J/kg.K
*C_s_*	Salt concentration, at the bulk, %
*de*	collision diameter of the water vapor and air, m^2^
*d_h_*	Hydraulic diameter, m
*D*	Diffusivity coefficient, m^2^/s
*h*_v_	Latent heat of vaporization, J/kg
*h*_f_, *h*_p_, *h*_m_	Feed, permeate, and membrane heat-transfer coefficients, W/m^2^ K
*J_w_*	Mass flux, kg/m^2^h
*k_B_*	Boltzmann’s constant
*k*_m_	Membrane conductivity, W/m.K
*k_s_*	Solid-phase thermal conductivity, W/m.K
*k_g_*	Gas-phase thermal conductivity, W/m.K
*k_n_*	Knudsen number
*l*	Channel height, m
*L, ln*	Channel length, m
m	Mass flow rate, kg/h
$m_{w}$	distillate flow rate, kg/h
M_w_	Molecular weight
*Nu*	Nusselt Number
*n*	Number of membrane length divisions, i.e., the control elements
P_1_, P_2_	Vapor pressure at the feed and permeate membrane surfaces, Pa
P	Average membrane interface pressure, Pa
P_a_	Entrapped air pressure, Pa
PD	Membrane pressure multiplied by diffusivity, Pam^2^/s
Pr	Prandtl number
*r*	Membrane pore size, m
R	Ideal gas constant also flows rate ratio
*Re*	Reynold Number
*t*	Time
*T_h_*, *T_c_*	Feed (hot) and permeate (cold) temperatures, K
*T_hb_*, *T_cb_*	Feed (hot) and permeate (cold) bulk temperatures, K
*T_hm_*, *T_cm_*	Feed and permeate membrane temperatures, K
$T_{h_{out}},\;T_{h_{in}}$	Outlet and inlet hot feed temperatures, °C
$T_{c_{out}},\;T_{c_{in}}$	Outlet and inlet cold stream temperatures, °C
T	The average temperature, at the membrane interface; K
*U*	Overall heat-transfer coefficient, W/m^2^K
*v*	Channel volume, m^3^
Greek letters	
α	Tuning parameter
τ	Tortuosity
ρ	Water density, kg/m^3^
δ	Membrane thickness
ε	Porosity
λ	Mean free path, m
μ	Viscosity coefficient, Pa/s
Δx	Control volume
Subscript	
*i*	Control element, *i*
*c*	Cold stream
*h*	Hot stream

## Appendix A

The separation of water by DCMD is governed by the simultaneous mass and heat-transfer mechanisms. In this section, we highlight the algorithm for solving the mass and heat transport equations to determine $h_{v},\;h_{m},\;{T_{h}}_{m}\;and\;{T_{c}}_{m}$. The latter variables are required to solve the dynamic model. The following algorithm was developed, adopting our previous experience with modeling the MD process [43,44,45,46]. The following algorithm assumed the process, under steady state conditions.

Employing the existing bulk temperatures ($T_{h_{b}},\;T_{c_{b}}$) for the hot and cold channels, the heat-transfer coefficients of the film ($h_{f},\;h_{p}$) could be estimated by the Nusselt number, as follows [4]:

(A1) $$Nu=0.298Re^{0.646}Pr^{0.316}$$

where Re is Reynolds number and Pr is the Prandtl number.

${T_{h}^{0}}_{m}=T_{h_{b}}\;and\;{T_{c}^{0}}_{m}=T_{c_{b}}$ were set.

The vapor pressure, at the membrane interface, was calculated by the following [5]:

(A2) $$P_{1}=\exp\left(23.238-\frac{3841}{T_{hm}-45}\right)\left(1-C_{s}\right)\left(1-0.5C_{s}-10C_{s}^{2}\right)$$

(A3) $$P_{2}=\exp\left(23.238-\frac{3841}{T_{cm}-45}\right)$$

The membrane coefficient, *C_m_*, can be computed based on the active mechanism by the membrane properties and the average membrane temperature, i.e., $T=\frac{{T_{h}}_{m}+{T_{c}}_{m}}{2}$. The active mechanism was determined under the following conditions [12]:

The Knudsen flow mechanism, *k_n_* > 1:

(A4) $$C_{m}^{k}=\frac{2\varepsilon r}{3\tau \delta }{\left(\frac{8M_{w}}{\pi RT}\right)}^{1/2}$$

Molecular diffusion mechanism, *k_n_* > 0.01:

(A5) $$C_{m}^{D}=\frac{\varepsilon }{\tau \delta }\frac{PD}{P_{a}}\frac{M_{w}}{RT}$$

Knudsen–molecular diffusion transition mechanism, 0.01 < *k_n_* < 1:

(A6) $$C_{m\;}^{C}={\left[\frac{3}{2}\frac{\tau \delta }{\varepsilon r}{\left(\frac{\pi RT}{8M_{w}}\right)}^{1/2}+\frac{\tau \delta }{\varepsilon }\frac{P_{a}}{PD}\frac{RT}{M_{w}}\right]}^{-1}$$

where the Knudsen number, defined as $k_{n}=\frac{\lambda }{d}$, and where λ is the mean free path of water molecules, further expressed as Equation (A7) [4]:

(A7) $$\lambda =\frac{k_{B}T}{\sqrt{2}\pi Pd_{e}{}^{2}}$$

where *T* and *P* are the average temperature and pressure, at the membrane interface, respectively, *k*_B_ = 1.380622 × 10^−23^ and *d*_e_ = 9.29 × 10^−20^.

The latent heat of vaporization, at the average membrane temperature, was calculated by the following equation [47]:

(A8) $$h_{v}\left(T\right)=1850.7+2.8273T-1.6\times 10^{-3}T^{2}$$

The mass flux was computed by the following equation:

(A9) $$j_{w}=C_{m}\left(P_{1}-P_{2}\right)$$

The overall heat-transfer coefficient was calculated, as follows [12]:

(A10) $$U={\left[\frac{1}{h_{f}}+\frac{1}{h_{m}+\frac{J_{w}h_{v}}{T_{h_{m}}-T_{c_{m}}}}+\frac{1}{h_{p}}\right]}^{-1}$$

where *h_m_* is the heat-transfer coefficient of the membrane, which involves the conduction resistance. It is computed, as follows [17]:

(A11) $$h_{m}=\frac{k_{m}}{\delta }=\frac{\left(1-\varepsilon \right)k_{s}+{\varepsilon k}_{g}}{\delta }$$

At a steady state, the different heat-transfer mechanisms become equal. These equalities translate into the following expressions [12]:

(A12) $$U\left(T_{h_{b}}-T_{c_{b}}\right)=h_{f}\left(T_{h_{b}}-T_{h_{m}}\right)=j_{w}h_{v}+h_{m}\left(T_{h_{m}}-T_{c_{m}}\right)$$

(A13) $$U\left(T_{h_{b}}-T_{c_{b}}\right)=h_{p}\left(T_{c_{m}}-T_{c_{b}}\right)=j_{w}h_{v}+h_{m}\left(T_{h_{m}}-T_{c_{m}}\right)$$

The above equalities can be solved to calculate the new quantities of $T_{h_{m}}\;and\;T_{c_{m}}$. If $T_{h_{m}}={T_{h}^{0}}_{m}\;and\;T_{c_{m}}={T_{c}^{0}}_{m}$, the iteration should be stopped. If not, set ${T_{h}^{0}}_{m}=T_{h_{m}}\;and\;{T_{c}^{0}}_{m}=T_{c_{m}}$ and go back to step 3.

The above algorithm is terminated with a termination tolerance of $1\times 10^{-7}$.
